# Near-infrared fluorescence laparoscopy of the ureter with three preclinical dyes in a pig model

**DOI:** 10.1007/s00464-018-6596-z

**Published:** 2018-11-26

**Authors:** Jacqueline van den Bos, Mahdi Al-Taher, Nicole D. Bouvy, Laurents P. S. Stassen

**Affiliations:** 1 Maastricht University Medical Center, Maastricht, The Netherlands; 2 NUTRIM, Maastricht University, Maastricht, The Netherlands; 3 Zuyderland Medisch Centrum, Heerlen, The Netherlands

**Keywords:** Fluorescent dyes, laparoscopic colorectal surgery, Near-infrared fluorescence imaging, Ureter

## Abstract

**Background:**

Ureteric injury is reported to occur in 1–7.6% of colorectal surgeries. To reduce the incidence of ureteral injury, it is essential to identify the ureters. The use of near-infrared fluorescence (NIRF) imaging with intravenously administered dyes might be of added value for ureteral visualization during laparoscopy. The aim of this study is to assess the performance of three preclinical dyes; IRDye^®^ 800BK, IRDye^®^ 800NOS and IRDye^®^ 800CW, for near-infrared fluorescence laparoscopy of the ureter in pigs.

**Methods:**

In three female Dutch landrace pigs, the new dyes were evaluated. In each pig, 1 dye was tested using a 6-mg intravenous dose in a concentration of 1 mg/ml. Imaging was performed in fluorescence mode and white light mode with a laparoscopic imaging system. In order to further evaluate the dyes, an ex vivo imaging experiment was performed, in which 8 decreasing concentrations per dye, diluted in PBS, were evaluated in a transparent test tube with NIRF mode at a distance of 1, 5 and 10 cm from the laparoscope.

**Results:**

All three dyes were effective in allowing the identification of the ureter with NIRF imaging. The ureter became fluorescent after 35, 45 and 10 min, respectively, for IRDye^®^ 800BK, IRDye^®^ 800NOS and IRDye^®^ 800CW with a maximum target-to-background ratio (TBR) of 2.14, 0.66 and 1.44, respectively. In the ex vivo imaging experiment, all three dyes produced a strong fluorescence signal at all concentrations and all distances evaluated.

**Conclusions:**

Intravenous administration of the preclinical dyes IRDye^®^ 800CW, IRDye^®^ 800 BK and IRDye^®^ 800NOS facilitated successful identification of the anatomical course of the ureter in living pig models. The highest measured TBR occurred with the use of IRDye^®^ 800BK. Ex vivo, a correlation was observed between the fluorescence intensities of the signal with the concentration of the dye and with the distance to the object.

With a reported incidence rate of 1% up to 7.6%, the occurrence of ureteric injury is one of the feared complications in colorectal surgery [1–4]. Ureteric injury can result in pain, intra-abdominal sepsis, systemic infection, abscesses, urinoma, ureteral stricture, ureteric fistula, renal failure and loss of the ipsilateral renal unit [4–6].

Failure to identify the relevant anatomy seems to be the main factor leading to ureter damage [7]. To avoid ureteric injury, it is therefore essential to identify both ureters during surgery. However, this may be difficult and time-consuming. Ureter stent placement is a technique which can be used in open surgery to help identify the ureters [4]. In laparoscopic surgery, however, in which tactile feedback is limited, this application is hardly useful. A technique improving the visualization of ureters in laparoscopic surgery is therefore needed. The use of near-infrared fluorescence (NIRF) imaging might be able to meet this need. This technique is already used in hepatobiliary surgery to visualize the bile ducts [8] or the perfusion of the liver [9], and in colorectal surgery to assess the perfusion of the bowel anastomosis [10–15]. A few studies have also been published on the use of NIRF imaging to identify the ureters.

The most commonly used fluorescent dye in laparoscopic surgery is indocyanine green (ICG). However, administering this dye intravenously does not facilitate the visualization of the ureters, as it is exclusively cleared by the liver, and therefore not excreted via the urine [16]. Alternatively, ICG might have potential for visualizing the ureters if administered through a ureter stent [17, 18]. Disadvantages of this technique include its invasiveness, the requirement of cystoscopy and possible complications such as urinary tract infections, hydronephrosis and hematuria [4]. This indicates that the use of ICG is a less than optimal option for the visualization of ureters during laparoscopy.

The above illustrates that there is a need for a potent fluorescent dye that can be administered intravenously, is cleared by the kidneys and has no side effects. A previous study showed that the preclinical IRDye^®^ 800CW (LI-COR Inc., Lincoln, NE, USA) provided clear ureter visualization in a porcine model [19] and, as such, has the potential to be such a dye. As this dye is quite expensive for this application, the manufacturer of IRDye^®^ 800CW developed two new, less expensive dyes: IRDye^®^ 800BK and IRDye^®^ 800NOS. These dyes proved to be very promising in cystic duct and cystic artery visualization after intravenous administration [20]. They are both partially cleared by the liver and by the kidneys, which makes them eligible to be used in NIRF imaging of the ureters after intravenous administration.

The aim of this study is to assess the performance of IRDye^®^ 800BK, IRDye^®^ 800NOS and IRDye^®^ 800CW in visualizing the ureter in pigs during near-infrared fluorescence laparoscopy.

## Materials and methods

This study was conducted at the central animal facilities of Maastricht University (Maastricht, The Netherlands). Animals were used in compliance with the regulations of the Dutch legislation concerning animal research, and the study was done according to a protocol that was approved by the local animal ethics committee. A pig model was chosen because of the similarities between pig anatomy and human anatomy and because of earlier successful application of NIRF imaging in pigs [19]. The experiments were done in three female Dutch landrace pigs (each weighing 40 kg).

### Laparoscopic fluorescence imaging system

A laparoscopic fluorescence imaging system (Karl Storz GmbH &CO. KG, Tittlingen, Germany) with a xenon-based light source was used. This system enables excitation and detection of all three dyes used in this experiment: IRDye 800CW (ʎEX/EM = 775/796 nm), IRDye^®^ 800BK (ʎEX/EM = 774/790 nm) and IRDye^®^ 800NOS (ʎEX/EM = 767/786 nm). All procedures were digitally recorded with the built-in recording equipment. The same NIRF imaging settings were used for all three dyes tested.

### Characteristics and preparation of the dyes

IRDye^®^ 800CW is a tetrasulphonated heptamethine indocyanine dye. After intravenous injection, it is cleared by the kidneys and excreted into urine. It is also partially cleared by the liver and excreted into bile. It can therefore be used for both bile duct and ureter visualization. Its maximum absorption occurs at 775 nm and its maximum excitation emission at 796 nm. The molecular weight of IRDye^®^ 800CW is 1090.11 Da [21].

IRDye^®^ 800BK and IRDye^®^ 800NOS are two newly developed dyes. IRDye^®^ 800BK is a hydrophilic dye with a maximum absorption of 774 nm and a maximum emission of 790 nm. Because of its hydrophilic nature, it is primarily cleared by the kidneys. This makes the dye especially useful for intraoperative ureter imaging. Nevertheless, some clearance by the liver takes place.

IRDye^®^ 800NOS is less hydrophilic and, as such, primarily cleared by the liver. This theoretically makes it especially useful for visualization of the biliary system. However, this dye is also partially excreted by the kidneys and could therefore also facilitate the visualization of ureters. The maximum absorption occurs at 767 nm and its maximum emission at 786 nm.

The dyes were prepared and used following instructions of the manufacturer.

### Surgical technique and assessment guidelines

The surgical procedures were performed under general anesthesia, as has been previously described in an earlier study [20]. Surgical residents performed a laparoscopic partial excision of the bicornate uterus, mimicking a laparoscopic appendectomy. These procedures were strictly supervised by two expert endoscopic gastrointestinal surgeons (NB and LS). Each pig received one of the three dyes intravenously at a total dose of 6 mg (1 mg/ml).

Imaging was performed intermittently in fluorescence mode and white light mode. Intraoperatively, the first authors (MA and JvdB) systematically documented whether the ureters could be identified in fluorescence mode, by filling in a registration form. The attending surgeon was consulted to reach agreement on the identification of the ureters. A structure was defined as ‘identified’ if its localization was confirmed with certainty by the experienced surgeon.

### Ex vivo NIRF imaging

In the ex vivo experiment, 8 decreasing dye concentrations, diluted in PBS, were evaluated in NIRF mode, in a completely darkened room with the laparoscope held at a distance of 1, 5 and 10 cm, respectively. A transparent 10 ml test tube was filled with 10 ml of each dye concentrations. The initial dilution consisted of 10 mg of the dye being diluted in 10 ml of PBS. From this basic concentration, decreasing dye concentrations were made. The evaluated concentrations of dye/PBS were 1:1, 1:2, 1:4, 1:8, 1:16, 1:32, 1:64 and 1:128.

### Postoperative analysis of the fluorescence

For an objective assessment of the fluorescence illumination, OSIRIX Lite v9 imaging software (Pixmeo, Geneva, Switzerland) was used. Fluorescence was analyzed by determining the fluorescence intensity (FI) and the target-to-background ratio (TBR). By doing so, it could be objectified whether the target organ (the ureter) was more fluorescent compared to the surrounding tissues. The TBR is defined as the mean fluorescence intensity (FI in arbitrary units, A.U.) of three points of interest in the target (ureter), minus the mean fluorescence intensity of three points of interest in the background divided by the mean fluorescence intensity of three points of interest in the background. In formula: TBR = (FI of target − FI of background)/(FI background) [19, 20, 22]. Areas with light scattering were avoided in these points of interest. The background FI in the ex vivo experiments was negligible due to the completely darkened room, and therefore, no TBR was calculated.

For the FI of the target, three centrally located regions of interest were chosen in the ureter. The mean fluorescence intensity of these three regions was the FI of target. When using this technique to establish the TBR, choosing a reproducible background is important. Therefore, as background 3 regions of interest 1 cm bilateral (2 right and 1 left) from the ureter were chosen in the in-vivo study. The mean scores of these three fluorescence intensities were used as the FI of background.

For the ex vivo study, the mean of three regions of interest at the center of the test tube filled with dye dilution was chosen as a target.

## Results

In all three experiments, it was possible to successfully identify the ureters with NIRF imaging. Results obtained during the operations are presented in the intraoperative registration form in Table 1. A representative screenshot in fluorescence mode was made of each of the three pigs.

**Table 1:** Intraoperative registration form

	Injected dye	Visualization left ureter	Visualization right ureter	Time to first certain visualization of ureter	Highest measured TBR
1	IRDye^®^ 800BK	Yes	Yes	35 min	2.14
2	IRDye^®^ 800NOS	Yes	Yes	45 min	0.66
3	IRDye^®^ 800CW	Yes	Yes	10 min	1.44

The first witnessed identification of the ureters occurred within minutes after dye administration with all three dyes. The ureteral wall became fluorescent in NIRF mode together with the uterus, bowel and lymph nodes. No peristaltic movement of urine within the ureters could yet be seen. Therefore, we could not identify the course of the ureter with absolute certainty at this stage.

**Fig. 1:**
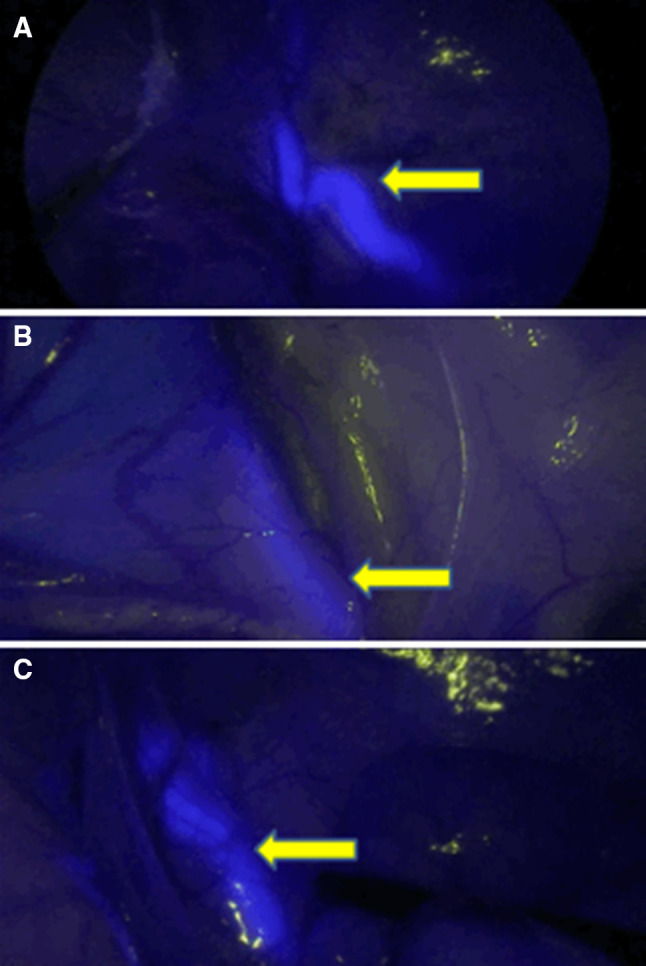
Visualization of the ureter with the experimental dyes: **a** with IRDye^®^ 800BK with highest TBR (2.14), **b** with IRDye^®^ 800NOS with highest TBR (0.66), **c** with IRDye 800CW with highest TBR (1.44)

Thirty-five minutes after the administration of the IRDye^®^ 800BK dye, peristaltic movement of the urine was clearly visible in NIRF mode due to the fluorescent dye excreted in the urine. The ureters remained fluorescent until the final assessment 3.5 h after dye administration. The highest measured TBR was 2.14 (Fig. 1a).

The first appearance of fluorescence of the ureters in the second pig (IRDye^®^ 800NOS) occurred 45 min after dye administration with clear peristaltic movement of urine within the ureters. The highest measured TBR was 0.66 (Fig. 1b).

In the third pig, the first fluorescent imaging of the ureters occurred 10 min after the IRDye^®^ 800CW dye was administered. Both ureters were clearly distinguishable from their surroundings. A second evaluation, 25 min after dye administration, showed a persistent clear delineation of the ureters in NIRF mode. The highest measured TBR was 1.44 (Fig. 1c).

No complications or adverse reactions were observed in any of the experiments.

### Ex vivo NIRF imaging

**Fig. 2:**
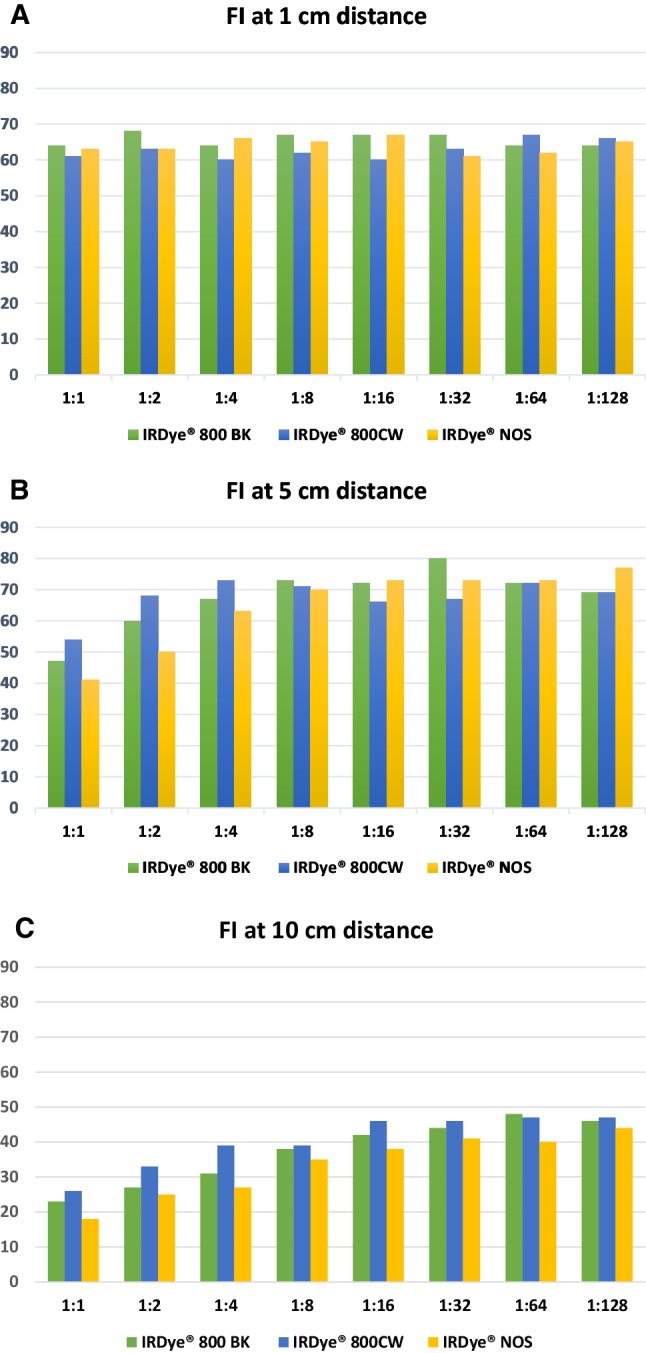
Fluorescence intensities (FI) for the three dyes for different dye concentrations with the laparoscope held at **a** 1 cm, **b** 5 cm and **c** 10 cm distance from the object. 1:1, 2:1, etc: dilutions of the initial dye concentration (see text)

With all three dyes, a strong fluorescence signal was achieved in all concentrations and at all distances evaluated. The results are depicted in Fig. 2a–c.

At 1 cm distance, all dyes in all concentrations showed comparable FI without clear fluctuations. At 5 cm distance, the FI of all dyes was lower in the highest concentrations. In these concentrations, IRDye^®^ 800CW showed the highest FI. This effect disappeared in the lower concentrations. At 10 cm distance, also an inverse relation between concentration and FI was observed for all three dyes. IRDye^®^ 800CW and IRDye^®^ 800BK showed a higher FI in all concentrations when compared to IRDye^®^ 800NOS. All three dyes showed a decrease in FI when the laparoscope was held at a 10 cm distance, as compared to when it was held at 1 and 5 cm distance, respectively. The highest FIs were achieved at 1 cm distance for the higher concentrations and at a 5 cm distance for the lower concentrations.

## Discussion

The most frequently used near-infrared fluorescent dye, indocyanine green, is exclusively cleared by the liver [20] and can therefore not be used for ureteral imaging after intravenous administration. Such imaging requires a dye that is excreted in urine or invasive techniques with a ureter catheter insertion through which the fluorescent dye can be introduced into its lumen [18, 23].

Verbeek et al. used methylene blue as a fluorescent dye for ureter visualization in open pelvic surgery in 12 patients. In all 12 patients, the ureters could be clearly visualized [24]. Yueng et al. showed visualization of the ureters in 10 out of 11 included patients. The technique was considered useful in 4 of these 10 cases. A recent study by Barnes et al. showed promising results allowing visualization of the ureter in a clinical study in which fluorescence imaging with methylene blue was of added value compared to white light in 14 of 69 ureters assessed [25]. However, in another study that used methylene blue as a fluorescent dye in laparoscopic colorectal surgery, this technique was not found to be superior to conventional white light laparoscopy in any of the included cases [26]. Although no side effects are reported in these studies, a disadvantage of using methylene blue is that it comes with some potential side effects, such as a small risk of anaphylactic reaction [27] and the potential occurrence of vasoconstriction.

A promising near-infrared fluorescent dye that is cleared by the kidneys is IRDye^®^ 800CW [23]. However, this dye is expensive for this application. Newer preclinical dyes which are cheaper to produce are IRDye^®^ 800NOS and IRDye^®^ 800BK. The manufacturer estimates their cost to be in the range of commercially available ICG (personal communication). In the present study, the usefulness of three preclinical dyes for NIRF imaging of the ureters was explored. IRDye^®^ 800BK is especially developed for excretion via the urine, being hydrophilic in nature. It is therefore expected that this dye will equal or outperform the imaging capabilities that have previously been described for IRDye^®^ 800CW [20].

All three dyes enabled clear and satisfactory visualization of the anatomical course of both ureters, as can be seen in Fig. 1a–c. The highest maximum in-vivo FI and TBR was measured with the IRDye^®^ 800BK, and the lowest maximum FI and TBR was found with the IRDye^®^ 800NOS. This is in line with expectations, as the hydrophilic IRDye^®^ 800BK is mainly cleared by the kidneys, while IRDye^®^ 800NOS is less hydrophilic and only partially cleared by the kidneys. Nevertheless, all three dyes give clear visibility of the ureters. The TBRs for IRDye^®^ 800CW were comparable with previous experiments [19].

In the ex vivo study, a clear fluorescence signal was achieved with all concentrations and at all the tested distances. The highest FIs were achieved when the laparoscope was held at 1 and 5 cm distance, whereas the lowest FI was achieved at a distance of 10 cm. This supports earlier studies which showed that the FI is negatively influenced by an increase in the distance of the laparoscope from the target [28].

Further studies evaluating more concentrations and distances may be helpful in identifying the ideal distance/concentration combination.

There were no adverse reactions as a result of the administration of the dyes. A transient decrease in SpO_2_ oxygen saturation is known with the use of intravenous fluorescent dyes, temporarily resulting in falsely lower oxygen levels [29, 30]. An advantage of these new dyes over iodine containing ICG is that these dyes can also be used in patients with a known hypersensitivity to iodine or iodine allergy. Due to the low number of pigs assessed, no conclusion may yet be drawn regarding the safety of the dyes.

Despite the promising results, the findings of this study have to be interpreted with caution. Since each dye was only tested in-vivo at one specific dosage and each dye was only tested in one pig, further experiments are needed to determine optimal dosing and timing of the dyes which are dependent on the pharmacokinetic properties of the dyes. Furthermore, testing in human subjects should be awaited in order to assess the clinical value of the dyes.

## Conclusion

Intravenous administration of the preclinical dyes IRDye^®^ 800CW, IRDye^®^ 800BK and IRDye^®^ 800NOS allowed for successful NIRF identification of the course of the ureters in a live pig model. The use of IRDye^®^ 800BK resulted in the highest contrast between ureter and background.

Ex vivo, a correlation of the signal was observed with the concentration of the dye and with the distance to the object.
